# Identification of four new susceptibility loci for testicular germ cell tumour

**DOI:** 10.1038/ncomms9690

**Published:** 2015-10-27

**Authors:** Kevin Litchfield, Amy Holroyd, Amy Lloyd, Peter Broderick, Jérémie Nsengimana, Rosalind Eeles, Douglas F Easton, Darshna Dudakia, D. Timothy Bishop, Alison Reid, Robert A. Huddart, Tom Grotmol, Fredrik Wiklund, Janet Shipley, Richard S. Houlston, Clare Turnbull

**Affiliations:** 1 Division of Genetics and Epidemiology, The Institute of Cancer Research, London SM2 5NG, UK; 2 Section of Epidemiology and Biostatistics, Leeds Institute of Cancer and Pathology, Leeds LS9 7TF, UK; 3 Royal Marsden NHS Foundation Trust, London SM2 5NG, UK; 4 Cancer Research UK, Genetic Epidemiology Unit, Strangeways Research Laboratory, Cambridge CB1 8RN, UK; 5 Academic Radiotherapy Unit, Institute of Cancer Research, Sutton, Surrey SM2 5NG, UK; 6 Department of Research, Cancer Registry of Norway, 0369 Oslo, Norway; 7 Department of Medical Epidemiology and Biostatistics, Karolinska Institutet, 171 77 Stockholm, Sweden; 8 Divisions of Molecular Pathology and Cancer Therapeutics, The Institute of Cancer Research, London SM2 5NG, UK; 9 William Harvey Research Institute, Queen Mary University, London EC1M 6BQ, UK

## Abstract

Genome-wide association studies (GWAS) have identified multiple risk loci for testicular germ cell tumour (TGCT), revealing a polygenic model of disease susceptibility strongly influenced by common variation. To identify additional single-nucleotide polymorphisms (SNPs) associated with TGCT, we conducted a multistage GWAS with a combined data set of >25,000 individuals (6,059 cases and 19,094 controls). We identified new risk loci for TGCT at 3q23 (rs11705932, *TFDP2, P*=1.5 × 10^−9^), 11q14.1 (rs7107174, *GAB2*, *P*=9.7 × 10^−11^), 16p13.13 (rs4561483, *GSPT1*, *P*=1.6 × 10^−8^) and 16q24.2 (rs55637647, *ZFPM1*, *P*=3.4 × 10^−9^). We additionally present detailed functional analysis of these loci, identifying a statistically significant relationship between rs4561483 risk genotype and increased *GSPT1* expression in TGCT patient samples. These findings provide additional support for a polygenic model of TGCT risk and further insight into the biological basis of disease development.

## Abstract (web summary)

This genome-wide association study identifies four novel risk loci for testicular germ cell tumour, and provides functional correlation between a disease-associated variant and gene expression in patient samples for one of the identified loci.

## Graphical abstract

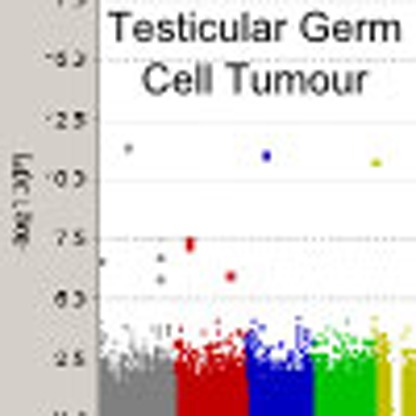

Testicular germ cell tumour (TGCT) is the most common cancer in men aged 15–45 years, with over 18,000 new cases diagnosed annually in Europe^1^,^2^. The incidence of TGCT has approximately doubled over the last four decades in Western Europe^3^, which implicates environmental or lifestyle factors as risk determinants. However, to date, no exogenous associations have been robustly validated^4^. Family and twin studies support a strong genetic basis to TGCT susceptibility^5^,^6^, with brothers of cases having an eight-fold increased risk of TGCT^7^. Direct evidence for inherited genetic susceptibility to TGCT has come from recent genome-wide association studies (GWAS), which have identified a number of independent loci influencing TGCT risk^8^,^9^,^10^,^11^,^12^,^13^,^14^,^15^,^16^,^17^. The associations identified by GWAS have provided novel insights into the development of TGCT, highlighting the role of genes involved in *KIT/KITLG* signalling, telomerase function, microtubule assembly and DNA damage repair^18^.

The over-representation of association signals in GWAS after accounting for known risk loci supports the existence of additional risk loci for TGCT. To identify new risk variants for TGCT, we have performed a GWAS meta-analysis, genome-wide imputation and large scale replication genotyping. Our combined data set comprises over 25,000 individuals and >8 million single-nucleotide polymorphism (SNPs), the largest study of its kind to date for TGCT. We report the identification of four new risk loci for TGCT.

## Results

### Association analyses

**Figure 1:**
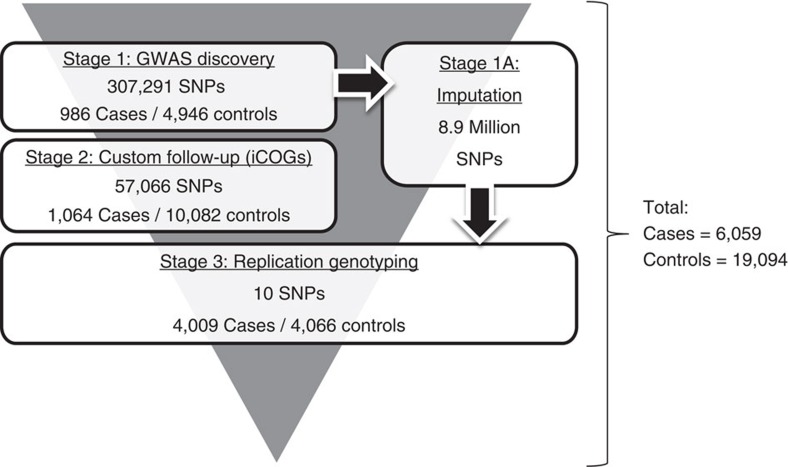
Study design, genotyping conducted over three stages, comprising non-overlapping samples from the UK. Imputation was performed on stage 1 GWAS data set.

We adopted a three-stage design, incorporating GWAS discovery, custom array follow-up and replication genotyping (Fig. 1). Genome-wide discovery (stage 1) was performed in 986 TGCT cases and 4,946 controls for 307,291 SNPs, as previously described^10^,^16^. The most strongly associated SNPs from stage 1 were included on a custom consortia array (iCOGs) and follow-up genotyping (stage 2) was conducted in an additional 1,064 cases of TGCT and 10,082 controls, as previously described^12^,^19^. Meta-analysis was then conducted on 57,066 SNPs overlapping between stages 1 and 2. To achieve dense genome-wide coverage, we retrospectively imputed unobserved genotypes (stage 1a) using our discovery GWAS data set and the 1000 genomes project reference panel. Results from meta-analysis and imputation were filtered to identify 20 SNPs at 12 loci with promising signs of association on the basis of the following criteria: (i) *P*<5.0 × 10^−4^, (ii) SNPs mapping to distant loci not previously associated with TGCT risk, (iii) *in silico* look-up in a Scandinavian GWAS data set comprising 1,326 cases and 6,687 controls genotyped using Human OmniExpressExome-8v1 Illumina arrays (*P*<0.1, ref. 17), (iv) consistent odds ratio (OR) effect sizes and allelic frequencies across all data sets. For these 12 loci, we conducted a replication study (stage 3), genotyping an additional 4,009 TGCT cases and 4,066 controls. Genotyping was successful for SNPs at 10 of the 12 loci. All the case and control samples were from the UK and formed unique sets, with no individuals overlapping between stages.

**Figure 2:**
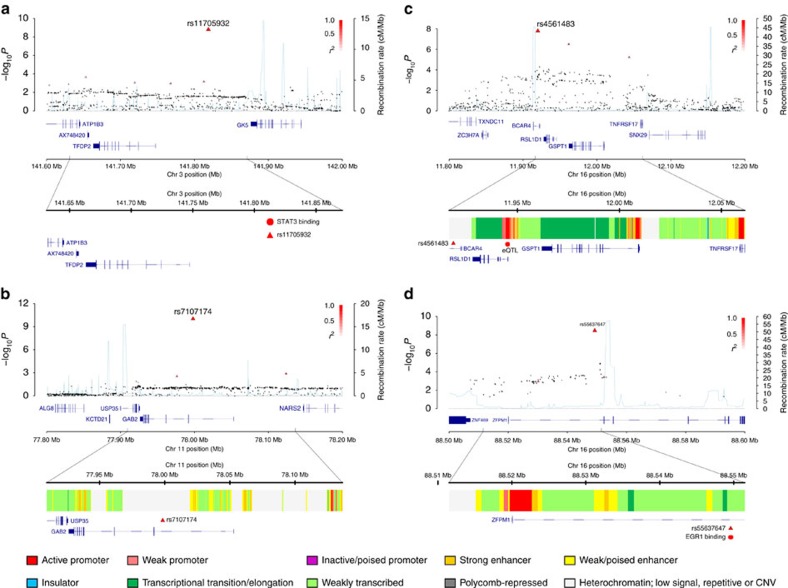
Regional plots of the four new TGCT loci. (**a**–**d**) Shown by triangles are the −log_10_ association *P* values of genotyped SNPs, based on meta-analysis (three-stage data for sentinel SNPs) and stages 1/2 for all other SNPs. Shown by circles are imputed SNPs at each locus, which were imputed from the stage 1 data set. The intensity of red shading indicates the strength of LD with the sentinel SNP (labeled). Also shown are the SNP build 37 coordinates in mega-bases (Mb), recombination rates in centi-morgans (cM) per mega-base (Mb) (in light blue) and the genes in the region (in dark blue). The zoomed-in section displays the exact LD block for each SNP, with the sentinel SNP marked with a red triangle, any significant regulatory markers denoted with a red circle and the chromHMM prediction states coloured as per the legend.

We tested association between each SNP and TGCT risk at each stage using the 1 d.f. trend test, with data from stages 1 and 2 being adjusted for six principal components. Inflation in the test statistics was observed at only modest levels (*λ*<1.05, *λ*_1000_<1.02 across all the stages). A combined fixed-effects meta-analysis was performed for SNP data across all the stages, for the 10 successfully genotyped loci. In the combined meta-analysis, SNPs at four novel loci attained genome-wide significance (*P*<5.0 × 10^−8^; Table 1, Fig. 2). First, rs11705932 (OR=1.18, confidence interval (CI)=1.09–1.28, *P*=1.5 × 10^−9^) which lies within a 240 kb region of linkage disequilibrium (LD) at 3q23, containing genes *TFDP2* and *ATP1B3*. Second, rs7107174 (OR=1.26, CI=1.16–1.37, *P*=9.7 × 10^−11^) which maps to intron 1 of *GAB2* (11q14.1), in a 227 kb region of LD to which *USP35* also localizes. Third, rs4561483 (OR=1.09, 95% CI=1.02–1.16, *P*=1.6 × 10^−8^) intronic to *BCAR4* (16p13.13) within a 145 kb LD block also containing *RSL1D1, GSPT1* and *TNFRSF17*. Finally, rs55637647 (OR=1.17, CI=1.09–1.24, *P*=3.4 × 10^−9^) mapping within intron 1 of *ZFPM1* (16q24.2), within a 40 kb LD block.

We examined for evidence of genotype-specific effect for rs11705932, rs7107174, rs4561483 and rs55637647, however, no significant departure from a log-additive model was seen. We additionally tested for interaction between rs11705932, rs7107174, rs4561483 and rs55637647 and SNPs at previously identified risk loci for TGCT (Supplementary Table 2). Some evidence of interaction between rs11705932 and previously reported SNP rs12699477 (at 7p22.3) was shown (*P*=0.003), albeit nonsignificant after correcting for 84 tests.

### Functional analysis of the four new TGCT SNPs

To gain insight into the biological basis of associations at rs11705932, rs7107174, rs4561483 and rs55637647, we conducted expression quantitative trait loci (eQTL) analysis using RNA-seq expression and Affymetrix 6.0 SNP/exome sequencing data on 150 TGCT patients, which is publicly available through the cancer genome atlas (http://cancergenome.nih.gov/). Where the data for our sentinel SNP was not available, we analysed data for the best two proxy SNPs (defined as those with the highest *r*^*2*^ correlation) for which data were available, namely: 3q23 (sentinel SNP rs11705932), 11q14.1 (rs2450140, *r*^*2*^=0.88 and rs11237477, *r*^*2*^=0.86), 16p13.13 (rs2075158, *r*^*2*^=0.78 and rs2018199, *r*^*2*^=0.79) and 16q24.2 (rs3859027, *r*^*2*^=0.91 and rs12597021, *r*^*2*^=0.87). Each of the nine genes (*ATP1B3, BCAR4, GAB2, GSPT1, RSL1D1*, *TFDP2, TNFRSF17, USP35* and *ZFPM1*) within the LD blocks at the four new risk loci were tested for evidence of an eQTL. No significant associations were identified at 11q14.1, 3q23 or 16q24.2. However, a statistically significant association was found at 16p13.13, between genotype and expression of *GSPT1* (proxy SNPs rs2075158 *P*=5.1 × 10^−4^, rs2018199 *P*=5.9 × 10^−4^), which remained significant after correction for multiple testing (Supplementary Table 1). Both SNPs rs2075158 and rs2018199 can be considered good proxy markers, having high *r*^*2*^ correlation with and closely comparable minor allelic frequencies to, the sentinel SNP. Homozygosity for the risk allele at rs2075158 was associated a with 35% increase in *GSPT1* expression compared with the reference homozygote genotype (Supplementary Fig. 1).

We used HaploReg^20^ and Roadmap Epigenome Mapping Consortium data on enhancer elements to examine whether rs11705932, rs7107174, rs4561483 and rs55637647 or their proxies (that is, *r*^*2*^>0.8 in 1000 Genomes CEU reference panel) lie at putative transcription factor binding/enhancer elements. In addition, we analysed GERP (Genomic Evolutionary Rate Profiling) scores to assess sequence conservation (Supplementary Data). At 11q14.1, which contains *GAB2*, there is evidence of strong evolutionary conservation, with 21 correlated SNPs having GERP score >2.0, the strongest of which is SNP rs2511156, which is in almost perfect LD with the sentinel SNP. In addition, multiple correlated SNPs at 11q14.1 are predicted to be in strong enhancer regions, with four SNPs located within DNase hypersensitivity sites in the TGCT specific cell line NT2-D1. Furthermore, 10 correlated SNPs at 11q14.1 alter the binding motif of embryonic transcription factor *NANOG*, a pluripotency factor strongly implicated in TGCT development^21^. At 16q24.2, the sentinel SNP rs55637647 is conserved and EGR1 binding, an early growth response transcription factor linked to infertility and differential expression in germ cell tumours^22^,^23^, was also reported within the LD block. No evidence of evolutionary conservation was seen for any SNPs at either 3q23 or 16p13.13 risk loci; however, both loci feature SNPs mapping to predicted enhancers. In addition, the significantly associated eQTL SNP at 16p13.13 (rs2075158) lies within a predicted strong active promoter site. Both 3q23 and 16p13.13 risk loci also have SNPs shown to alter the binding motif of *SOX* family transcription factors, which regulate germ cell development and sex determination. In addition, the protein STAT3, which is critical for embryonic development and is expressed in the developing spermatids of adult testis^24^, binds to the locus at 3q23.

Finally, using matched tumour/normal exome sequencing data from our recent study of 42 UK TGCT patients^25^, we analysed somatic mutational events occurring in genes *ATP1B3, BCAR4, GAB2, GSPT1, RSL1D1*, *TFDP2, TNFRSF17, USP35* and *ZFPM1*. The only recurring event, seen in >5% of tumours was a copy number deletion encompassing *GAB2* and *USP35* at 11q14.1 found in 7% of tumours. These deletions were large, spanning up to 55 Mb.

### Pathway analysis

We performed gene set enrichment analysis to determine whether any of the genes mapping to our four newly identified loci reside in pathways already enriched with TGCT SNPs. Using the i-GSEA4GWAS algorithm^26^ on stage 1 data, a total 31 pathways showed enrichment in the analysis of genome-wide association data for TGCT (FDR <0.1; Supplementary Table 3). Five pathways that were of note were those involved in sex determination, centrosome cycle, apoptosis, *KIT/KITLG* signalling and DNA damage repair, further substantiating existing evidence linking these gene sets to TGCT^17^,^18^,^27^. Focusing on these five pathways, genes at three of the new loci feature (see Supplementary Fig. 2). The first related pathway involves *GAB2* at 11q14.1, a member of the *GRB2*-associated binding protein (GAB) gene family, which associates with *KIT* forming a critical part of the *KIT/KITLG* signalling cascade^28^. The second related gene is *ZFPM1* at 16q24.2, linked to sex determination, with *ZFPM1* being shown to specify germ cell differentiation as sperm rather than oocytes in *Caenorhabditis elegans*^29^. Both *ZPFM1* and its paralogue *ZPFM2* regulate the activity of GATA family of transcription factors, which are abundantly expressed from the onset of human gonadal development and found in multiple cell lineages of the testis^30^,^31^. The third related gene is *GSPT1* at 16p13.13, which is a documented determinant of apoptosis^32^.

### Personalized risk profiling

**Figure 3:**
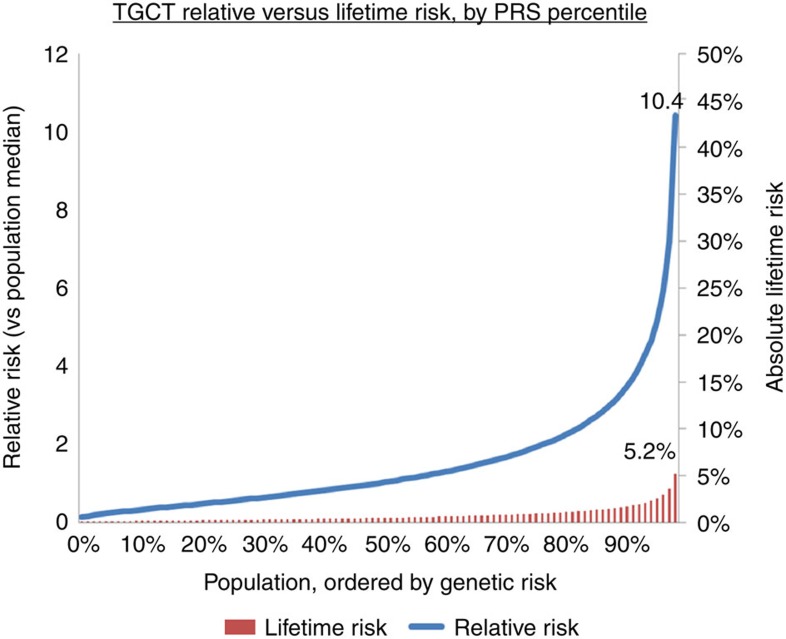
Population distribution of polygenic risk scores for TGCT, ordered from lowest to highest genetic risk (risk is relative to population median risk). Relative risk is plotted as a blue line, lifetime risk as red bars. Values are marked for individuals in the top 1% of highest genetic risk.

The OR effect sizes of TGCT SNPs have been among the highest reported in GWAS of any cancer type^33^, hence suggesting a potential clinical utility for personalized risk profiling. To assess this potential, we constructed polygenic risk scores (PRS) for TGCT, considering the combined effect of all risk SNPs modelled under a log-normal relative risk distribution, as implemented for other cancer types^34^,^35^,^36^. Using this approach for the four new risk loci, together with all existing risk SNPs (Supplementary Table 2), the men in the top 1% of genetic risk had a 10.4-fold relative and 5.2% lifetime risk of TGCT (Fig. 3).

## Discussion

Here we have genotyped the largest number of TGCT cases to date, identifying four novel TGCT susceptibility loci at 3q34, 11q14.1, 16p13.13 and 16q24.2. We additionally performed TGCT cell type-specific eQTL analysis of these loci, identifying a possible *cis*-regulatory effect on *GSPT1* expression at 16p13.13. Aside from the detailed functional work undertaken by Bond *et al*. exploring the mechanism underlying the signal at 12q21 (ref. 37), this is the first statistically significant eQTL identified for TGCT.

Of the four new loci, the functional mechanism at 16p13.13 is most tangible, with expression of *GSPT1* (G1- TO S-PHASE TRANSITION 1) found to be upregulated in risk allele carriers. *GSPT1* is a proto-oncogene essential for the G1-to-S phase cell cycle transition and regulates mammalian cell growth^38^,^39^. Perhaps not surprisingly, *GSPT1* has been shown to be upregulated in multiple tumour types, including cancers of the stomach, prostate and breast^40^,^41^,^42^. Furthermore, inherited variants in *GSPT1* have been reported to confer elevated risk of gastric cancer^41^. As the sample size of available RNA-seq expression data we used is relatively modest (*n*=150), the analysis of this effect in a larger data set would be of significant interest. *GSPT1* is also cited as a potential target for anticancer therapy^40^, due to its role regulating cell cycle progression, a process effectively targeted for various existing drug classes such as mTOR pathway inhibitors.

At the second locus (11q14.1), there are competing functional hypotheses, with strong TGCT cell type-specific evidence being observed to suggest an influence on gene expression. Of the two genes in LD at 11q14.1, a plausible candidate is *GAB2* (GRB2-associated binding protein 2), which encodes a docking protein that is important in signal transduction from tyrosine kinases and is bound by GRB2. *GAB2* has been demonstrated to act as a proto-oncogene in breast, colorectal and ovarian cancers as well as melanoma^43^,^44^, and has been shown to be therapeutically targetable by imatinib and dasatinib^45^. Our eQTL analysis did not demonstrate a link between rs7107174 and *GAB2* expression, although this failure may be due to the imperfect correlation between the true functional SNP and proxy markers available. Alternatively, other functional mechanisms may underpin the association; of particular note, a missense variant (rs2510044) responsible for the P236M polymorphism in *USP35* (ubiquitin-specific peptidase 35) is in perfect LD with our sentinel SNP. P236M is predicted to be pathogenic using the CONDEL algorithm^46^,^47^. In our somatic data sets, a recurring deletion encompassing both *GAB2* and *USP35* was found in 7% of tumours; however, due to the large scale of these deletions there is no evidence to suggest they specifically relate to the 11q14.1 locus.

At the third locus (16q24.2) *ZFPM1* (zinc finger protein, multitype 1, also known as *FOG, Friend of GATA1)* is the only gene in LD with the sentinel SNP. Although we cannot exclude a regulatory effect outside the LD block, *ZFPM1* provides an attractive functional basis for association being a regulator of the transcription factor *GATA1. ZFPM1* is expressed in human Sertoli cells, first in the late fetal stages and then throughout postnatal testicular development^48^. GATA transcription factors were first implicated in carcinogenesis over two decades ago, and their role in various types of leukaemia is now well established^49^. In addition, GATA1 directly contributes to the silencing of KIT, a pathway which is strongly implicated in both germline and somatic studies of TGCT^18^,^49^. The last remaining locus (3q23) contains genes *TFDP2* (Transcription Factor DP2) and *ATP1B3* (ATPase, Na^+^/K^+^ Transporting, Beta 3 Polypeptide). Although eQTL analysis was not able to establish a link between rs11705932 genotype and expression of either gene, *TFDP2* is a plausible functional candidate, as expression of this gene is itself regulated by binding of GATA1 (ref. 50). In this study, we therefore implicate *FOG/GATA1* genes in TGCT susceptibility for the first time, highlighting a network of interlinked oncogenic pathways.

These four new loci provide further biological insight into this tumour, as well suggesting a possible new target for TGCT therapy, with reduced toxicity potential compared with current treatment options. In addition, these loci add additional insights into the pathways relevant to TGCT susceptibility, in particular, to those related to sex determination, apoptosis and *KIT/KITLG* signalling. Our genome-wide pathway analysis also highlighted the centrosome cycle and DNA damage repair pathways, consistent with previous studies. More extensive pathway mapping of TGCT risk loci would be informative, in particular, to explore pathways related to telomerase function and male germ cell development. Both these latter two pathways are functionally related to genes in LD with existing TGCT risk loci (see Supplementary Table 2); however, they were not identified as significant by the iGSEA4GWAS algorithm, possibly owing to the imperfect nature of pathway definitions.

Our four new risk loci, together with the previously known risk SNPs for TGCT, collectively explain 19% of the sibling risk of TGCT. We constructed a PRS model to assess the clinical utility of TGCT risk SNPs, which demonstrated marked power in terms of risk discrimination, with men in the top 1% of genetic risk exhibiting a >10-fold increased risk of the disease. However consideration of lifetime risk highlights the rare nature of TGCT, with high relative risks translating into only modest absolute risk. Hence the current clinical utility of PRS-based risk stratification may be limited in terms of population level screening; however, targeted models (such as screening individuals at already elevated baseline risk) could offer more immediate benefit. In addition, discovery of additional risk SNPs may also improve clinical utility and recent population and genomic analyses of heritability have shown that: (i) TGCT is a highly heritable cancer (heritability ∼48%), and (ii) a significant proportion of the heritability is likely to reside within common SNPs^51^. It is therefore likely that additional GWAS and meta-analyses will indeed lead to the identification of further risk SNPs for TGCT.

In conclusion, by performing large-scale genotyping, we have identified four novel susceptibility loci for TGCT. Our functional analysis has identified a link between risk genotype at 16p13.13 and regulation of *GSPT1* expression, as well as highlighting plausible oncogenic candidates across the remaining loci.

## Methods

### Sample description

Cases with a diagnosis of TGCT were ascertained from two studies (1) a UK study of familial testicular cancer and (2) a systematic collection of UK collection of TGCT cases. Case recruitment was via the UK Testicular Cancer Collaboration, a group of oncologists and surgeons treating TGCT in the UK (Supplementary Note 1). The majority of cases included in stage 3 were sporadic (3,941 sporadic versus 68 familial), hence sub-analysis of sporadic versus familial effect size was not possible. The studies were co-ordinated at the Institute of Cancer Research (ICR). Samples and information were obtained with full informed consent and Medical Research and Ethics Committee approval (MREC02/06/66 and 06/MRE06/41).

Controls for the stage 1 GWAS were from two sources within the UK: 2,482 controls were from the 1958 Birth Cohort (1958BC) and 2,587 controls were identified through the UK National Blood Service (NBS) and were genotyped as part of the Wellcome Trust Case Control Consortium. Controls for the stage 2 genotyping were from three sources within the UK. Eight hundred and fourteen cancer-free, male controls age <65 from the UK were recruited through the UK Genetic Prostate Cancer Study (UKGPCS), a study conducted through the Royal Marsden NHS Foundation Trust. A total 7,871 cancer-free controls (1,244 male) were recruited via GP practices in East Anglia (2003–2009) as part of SEARCH (Study of Epidemiology & Risk Factors in Cancer). A total 1,397 cancer-free female controls from across the UK were recruited via the BBCS (British Breast Cancer Study). Controls for stage 3 replication genotyping were taken from two studies, the NSCCG (National Study of Colorectal Cancer Genetics)^52^ and the GELCAPS (GEnetic Lung CAncer Predisposition Study)^53^. The NSCCG and GELCAP controls were partners of cancer patients with no personal history of cancer at the time of ascertainment.

### Genotyping

Genotyping for stages 1 and 2 was performed as previously reported^10^,^12^,^16^. In brief, stage 1 cases were genotyped on the Illumina HumanCNV370-Duo bead array (Ilumina, San Diego, CA, USA) and controls were genotyped on the Illumina Infinium 1.2M array. We used data on 314,861 SNPs that were successfully genotyped on both the arrays. Stage 2 genotyping was conducted using a custom Illumina Infinium array (iCOGS array) comprising 211,155 SNPs selected across multiple consortia within the COGS (Collaborative Oncological Gene-environment Study), as previously described^12^,^19^. SNPs attaining an Illumina design score of ≥0.8 were included on the array. A total of 57,066 SNPs overlapped with our stage 1 data set and were included in the meta-analysis. For stage 3 genotyping, we designed KASPar allele-specific SNV primers^54^, genotyping 20 SNPs across the 10 loci. Genotyping was conducted by external laboratory LGC Limited, Unit 1–2 Trident Industrial Estate, Pindar Road, Hoddesdon, UK.

### Quality control

Stage 1 data were filtered as follows: we excluded individuals with (i) low call rate (<95%), (ii) abnormal autosomal heterozygosity or (iii) with >10% non-European ancestry (on the basis of multi-dimensional scaling). We filtered out all SNPs with (i) minor allele frequency <1%, (ii) a call rate of <95% in cases or controls or (iii) minor allele frequency of 1–5% and a call rate of <99% or (iv) deviation from Hardy–Weinberg equilibrium (10^−12^ in controls and 10^−5^ in cases). The final number of SNPs passing quality control filters was 307,291. Stage 2 data filtering were conducted on the full SNP set of 211,155 SNPs on the iCOGS array, with QC exclusions applied as follows to subjects using: (i) subjects with overall call rate <95% or deficit/excess of heterozygosity (*P*<10^−6^), (ii) using identity-by-state estimates based on 37,046 uncorrelated SNPs, we identified ‘cryptic' duplicates and related samples and the sample with the lower call rate was excluded, (iii) we identified ethnic outliers by multi-dimensional scaling by combining the iCOGS data with the three Hapmap2 populations using 37,046 uncorrelated markers and removed individuals with >10% non-Western European ancestry. We included 1,064 cases and 10,082 controls in the final analysis. Stage 2 QC was applied to the SNPs as follows: (i) discrepant calls in more than 2% of duplicate samples across COGS consortia, (ii) call rate <95%, MAF<1%, call rate <99% if MAF=1–5%, (iii) deviation from Hardy–Weinberg (*P*<10^−5^ in controls, *P*<10^−12^ in cases). For stage 3, of the 20 SNPs designed, 18 SNPs were successfully genotyped. From these 18 SNPs, one SNP from each of the 10 loci was selected, based on the strongest signal of association. The average call rate across the 10 selected SNPs was 99.1% with all SNPs having a call rate of >98.5%. All SNPs had a MAF >1% and no SNP deviated from HWE at *P*<0.1. Hence all 10 SNPs passed pre-specified QC metrics. Call rates were assessed for individuals in stage 3, with 99.1% of individuals achieving a call rate of ≥90% and 95.2% with call rate of 100%. A small number of individuals (*n*=32, 0.4%) failed across all 10 SNPs and were excluded from the analysis.

### Statistical analysis

Statistical analysis for stages 1 and 2 was performed as previously reported^10^,^12^,^15^,^16^. In brief, we tested for association between each SNP and TGCT risk at each stage using a 1 d.f. trend test, with data being adjusted for six principle components. Inflation in the test statistics was observed at only modest levels, with values before adjustment for principle components being: stage 1 inflation factor (*λ*)=1.08 (equivalent to *λ*_1000_=1.05) and stage 2 *λ*=1.14 (*λ*_1000_=1.07). After adjustment for principle components: stage 1 *λ*=1.00 (*λ*_1000_=1.00) and stage 2 *λ*=1.04 (*λ*_1000_=1.02). In stage 3, the 10 SNPs were tested for association with TGCT risk and per-allele ORs were estimated, using logistic regression with 1.d.f, in line with the stage 1 and stage 2 analyses. We obtained overall combined significance levels across all the three stages using a fixed-effects meta-analysis, using a threshold of *P*<5.0 × 10^−8^ to denote genome-wide significance. For each novel locus, we examined evidence of departure from a log-additive (multiplicative) model to assess any genotype-specific effect. Using stage 3 data, individual genotype data ORs were calculated for heterozygote (OR_het_) and homozygote (OR_hom_) genotypes, which were compared with the per-allele ORs. We tested for a difference in these 1 d.f. and 2 d.f. logistic regression models to assess for evidence of deviation (*P*<0.05) from a log-additive model. Using stage 1 data, we examined for statistical interaction between the four new loci and the existing 21 TGCT predisposition loci by evaluating the effect of adding an interaction term to the regression model, adjusted for stage, using a likelihood ratio test (using a significance threshold of *P*<5.95 × 10^−4^ to account for 84 tests). LD blocks were defined using the HapMap recombination rates (cM/Mb) and defined using the Oxford recombination hotspots^55^. Regional plots were generated using visPIG software^56^. PRSs were constructed using methods established by Pharoah *et al*.^57^, based on a log-normal distribution *LN* (*μ*, *σ*^2^) with mean *μ* and variance *σ*^2^ (that is, relative risk is normally distributed on a logarithmic scale). Lifetime TGCT risk was based on 2014 CRUK lifetime incidence rate of 0.5% (ref. 58), multiplied by RR to give lifetime risk per percentile of the PRS. Competing mortality risk analysis was not conducted as over three quarters of TGCT cases present at ages 45 years and younger^58^, for whom cumulative mortality risk from all other causes is only 3.6% (ref. 59).

### Imputation

Genome-wide imputation was performed using the genotyped data from stage 1. The 1000 genomes phase 1 data (September 13 release) was used as a reference panel, with haplotypes pre-phased using SHAPEIT2 (ref. 60). Imputation was performed using IMPUTE2 software^61^ and association between imputed genotype and TGCT was tested using SNPTEST^62^, under a frequentist model of association. QC was performed on the imputed SNPs; excluding those with INFO score <0.8 and MAF <0.01.

### Functional annotation

We used data from the ENCODE project and HaploReg^20^ to investigate for evidence of transcriptional regulation at our identified locus to assess (i) whether the variant resides in a region in which modification of histone proteins is suggestive of enhancer and other regulatory activity (H3K4Me1 and H3K27A histone modification) or promoter activity (H3K4Me3 histone modification), (ii) whether the variant lies in a region where the chromatin is hypersensitive to cutting by the DNase enzyme (suggestive of regulatory region), (iii) whether the variant lies in a region of binding of transcription factor proteins (as assayed by chromatin immunoprecipitation with antibodies specific to the transcription factor followed by sequencing of the precipitated DNA (ChIP-seq)), (iv) whether the variant affects a specific regulatory motif, as evaluated from position weighted matrices assembled from TRANSFAC, JASPAR and protein-binding microarray experiments.

We investigated for evidence of association between the SNPs at our locus and changes in gene expression using publicly available cancer genome atlas RNAseq and Affymetrix 6.0 SNP/exome sequencing data (http://cancergenome.nih.gov/). Where genotype data for our sentinel SNP was not available, we selected the top two closest proxy SNPs available in the combined SNP/exome data sets, based on highest *r*^2^ value. Associations between normalized RNA counts per gene and genotype were quantified using the Kruskal–Wallis trend test. A total of 18 tests were performed, hence a *P* value threshold of 0.0028 was considered significant to correct for multiple testing.

### Pathway analysis

Pathway enrichment analysis was conducted using the Improved Gene Set Enrichment Analysis for genome-wide association study (i-GSEA4GWASv1.1; ref. 26). Predefined biological pathways and processes including KEGG, reactome pathways and gene ontology gene sets (GO) were assessed for association with TGCT. SNPs within a ±5 kb distance were mapped to genes and the maximum −log(*P* value) of all the SNPs mapped to a gene was used to represent the gene, using SNP label permutation.

## Additional information

**Accession codes:** Stage 1 GWAS data have been deposited in the European Genome–phenome Archive (EGA), which is hosted by the European Bioinformatics Institute (EBI), under the accession code EGAS00001001302.

**How to cite this article:** Litchfield, K. *et al*. Identification of four new susceptibility loci for testicular germ cell tumour. *Nat. Commun.* 6:8690 doi: 10.1038/ncomms9690 (2015).

## Supplementary Material

Supplementary Information Supplementary Figures 1-2, Supplementary Tables 1-3, Supplementary Note 1 and Supplementary References

Supplementary Data 1 Genomic Evolutionary Rate Profiling analysis

## Figures and Tables

**Table 1:** Summary of results across all genotyping stages.

SNP*	**Chr.**	**Alleles**†	**RAF**‡	**Stage 1/1a—GWAS/Imputation**		**Stage 2—iCOGs**		**Stage 3—Replication**		**Combined**		
				**OR**§ **(95% CI)**	***P***_**trend**_||	**OR (95% CI)**	***P***_**trend**_	**OR (95% CI)**	***P***_**trend**_	***P***_**meta**_¶	***P***_**het**_#	***I***^**2**^ Het**
**rs11705932**	**3**	**T/****C**	**0.80**	**1.21 (1.07-1.37)**	**2.7 × 10**^−3^	**1.22 (1.08–1.38)**	**1.2 × 10**^**−3**^	**1.18 (1.09–1.28)**	**3.4 × 10**^**−5**^	**1.5 × 10**^**−9**^	**9.1 × 10**^**−1**^	**0**
rs147686985	3	G/C	0.02	1.80 (1.33–2.44)	2.6 **×** 10^**−**6^	—	—	1.06 (0.84–1.33)	6.4 **×** 10^**−**1^	4.0 **×** 10^**−**1^	9.4 **×** 10^**−**3^	85
rs13062518	3	T/C	0.43	1.21 (1.09–1.33)	2.6 **×** 10^**−**4^	1.14 (1.04–1.25)	6.1 **×** 10^**−**3^	1.06 (1.00–1.13)	6.3 **×** 10^**−**2^	9.6 **×** 10^**−**2^	1.0 **×** 10^**−**4^	91
rs16873802	5	T/C	0.03	1.76 (1.33–2.32)	3.0 **×** 10^**−**5^	—	—	1.06 (0.87–1.29)	5.4 **×** 10^**−**1^	2.9 **×** 10^**−**2^	1.1 **×** 10^**−**2^	85
rs6927322	6	T/G	0.04	1.55 (1.27–1.89)	1.2 **×** 10^**−**5^	—	—	1.24 (1.08–1.43)	3.2 **×** 10^**−**3^	6.1 **×** 10^**−**6^	1.1 **×** 10^**−**1^	61
rs13279707	8	T/C	0.05	1.58 (1.29–1.92)	7.5 **×** 10^**−**6^	1.28 (1.06–1.56)	1.1 **×** 10^**−**2^	1.06 (0.92–1.22)	4.2 **×** 10^**−**1^	2.7 **×** 10^**−**3^	1.0 **×** 10^**−**4^	89
**rs7107174**	**11**	**T/C**	**0.15**	**1.14 (1.01–1.30)**	**4.2 × 10**^**−2**^	**1.21 (1.07–1.36)**	**2.0 × 10**^**−3**^	**1.26 (1.16–1.37)**	**4.8 × 10**^**−8**^	**9.7 × 10**^**−11**^	**4.6 × 10**^**−1**^	**0**
**rs4561483**	**16**	**A/G**	**0.35**	**1.22 (1.10–1.35)**	**1.3 × 10**^**−4**^	**1.20 (1.10–1.32)**	**1.1 × 10**^**−4**^	**1.09 (1.02–1.16)**	**8.1 × 10**^**−3**^	**1.6 × 10**^**−8**^	**9.7 × 10**^**−2**^	**57**
rs3850997	16	T/G	0.33	1.17 (1.06–1.30)	2.5 **×** 10^**−**3^	1.18 (1.07–1.30)	7.6 **×** 10^**−**4^	1.06 (1.00–1.13)	6.9 **×** 10^**−**2^	1.0 **×** 10^**−**5^	1.2 **×** 10^**−**1^	54
**rs55637647**	**16**	**G/C**	**0.37**	**1.21 (1.10–1.34)**	**6.5 × 10**^**−5**^	—	—	**1.17 (1.09–1.24)**	**2.7 × 10**^**−6**^	**3.4 × 10**^**−9**^	**5.2 × 10**^**−1**^	**0**

SNPs highlighted in bold achieved genome-wide significance.

^*^dbSNP rs number.

^†^Alleles (risk allele is underlined).

^‡^Risk allele frequency.

^§^OR: per allele odds ratio.

^||^*P*_trend_: *P* value for trend, via logistic regression.

^¶^*P*_meta_: *P* value for fixed effects meta-analysis.

^#^*P*_het_: *P* value of heterogeneity between studies.

^**^I^2^ heterogeneity index (0–100).
