# Supplementary material for: Identification of four new susceptibility loci for testicular germ cell tumour
Source: Nat Commun. 2015 Oct 27;6:8690. doi: 10.1038/ncomms9690 (PMC4846317; doi:10.1038/ncomms9690)
Supplement: Supplementary Information — Supplementary Figures 1-2, Supplementary Tables 1-3, Supplementary Note 1 and Supplementary References [file ncomms9690-s1.pdf]

Supplementary Figures

Supplementary figure 1 – Boxplot of eQTL at 16p13.13. The dark black line represents the median expression value for each genotype and the box represents the upper and lower quartiles of data above/below the median. The whiskers extend out to 0.2 times the interquartile range.

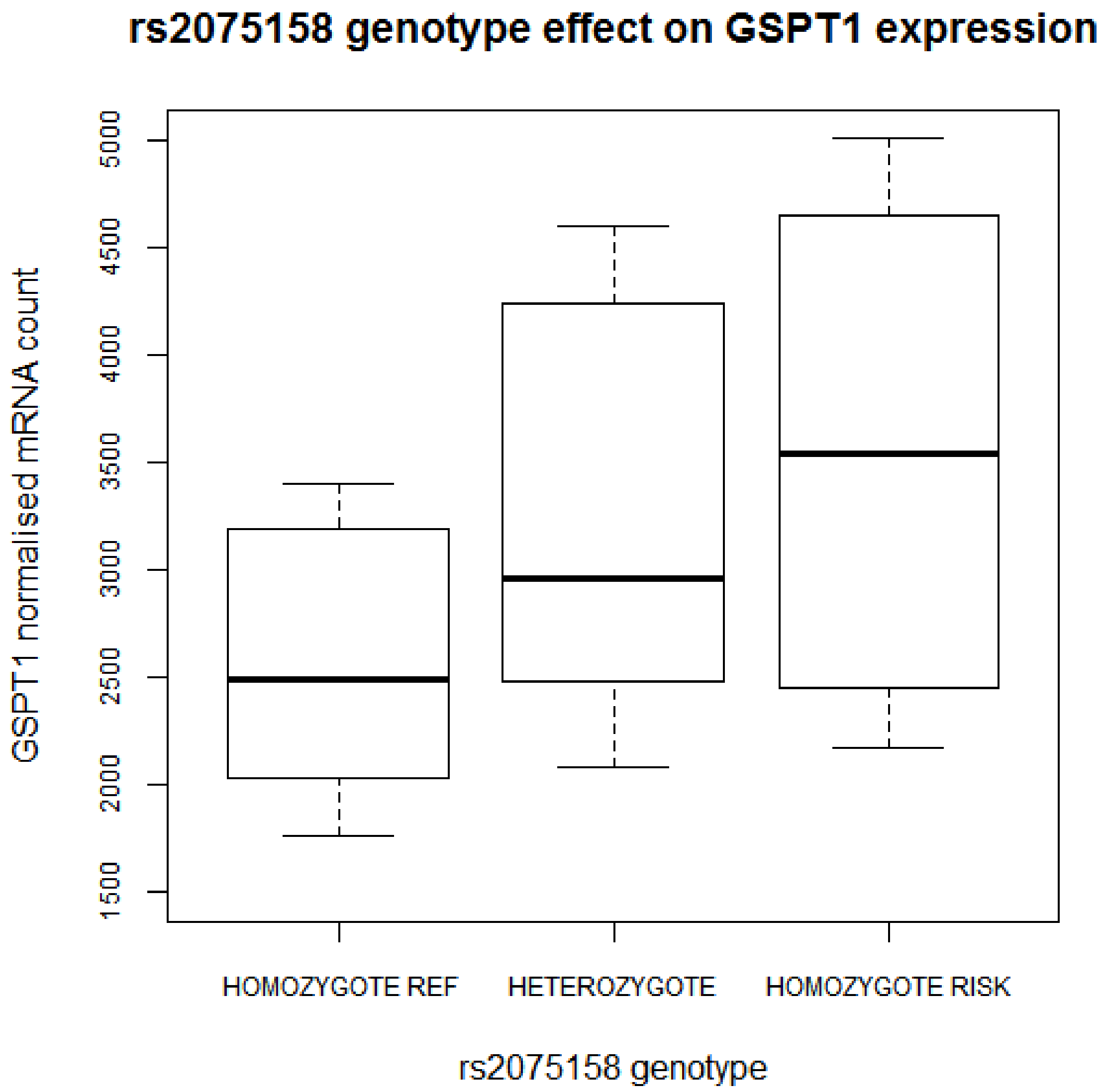

Supplementary figure 2 - Significantly enriched TGCT Pathways, implicated in this paper and at least one other study (references in brackets). Gene set names are in underlined black text, ordered with the most significant pathway first (Sex Determination) and then subsequent pathways presented in clockwise order with decreasing significance. The values on the blue line represent the false discovery rate for each pathway as calculated by the iGSEA4GWAS algorithm. In the text boxes are selected genes related to each pathway, colour-coded as follows: dark blue underlined text = genes in LD with risk SNPs identified in previous TGCT GWAS, red underlined text = genes in LD with the four new loci identified in this study, black plain text = genes not yet in LD with any TGCT risk SNPs but highlighted as significant by the iGSEA4GWAS algorithm. Pathways marked with an asterisk are custom gene sets, with "Sex determination" pathway defined as per [1] and the "KIT/KITLG" pathway defined based on known interacting partners of KIT/KITLG using the STRING database[2].

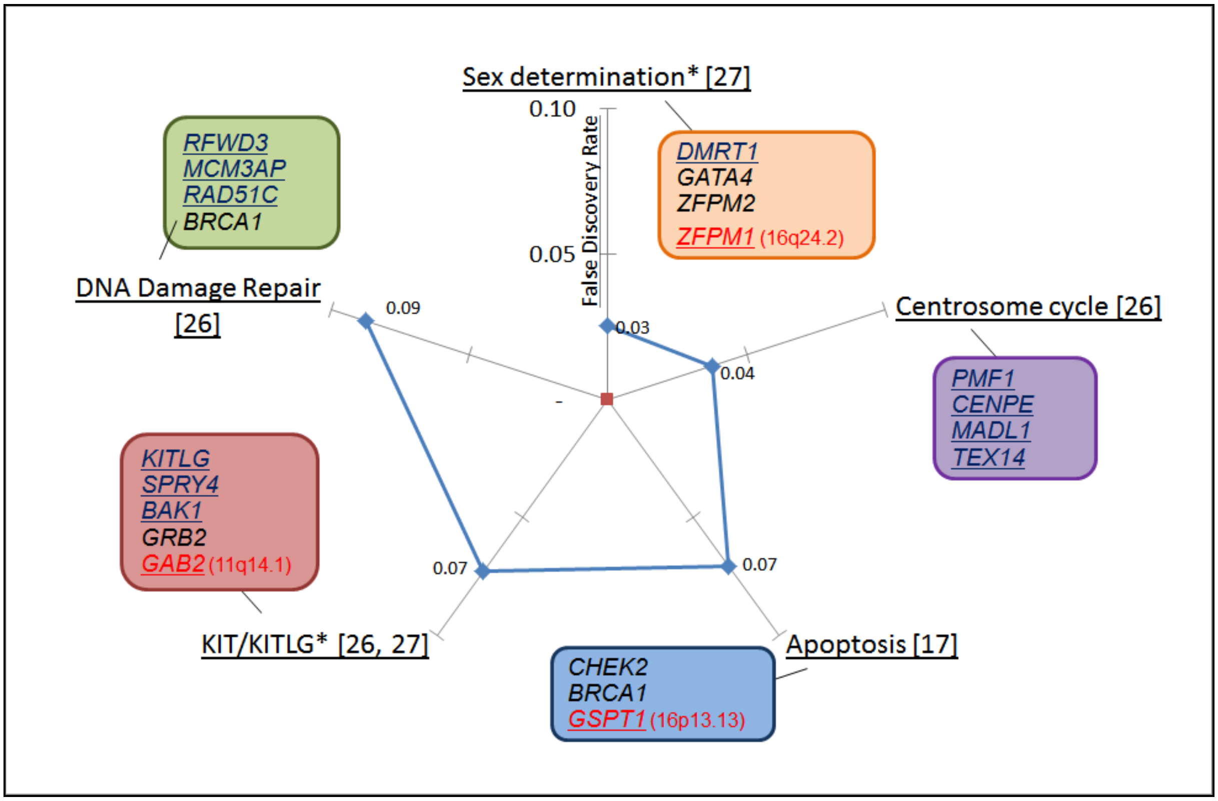

Supplementary Tables

Supplementary table 1 – *P*-values from eQTL tests performed. *P*-value threshold 0.0028 considered significant, corrected for 18 multiple tests.

	11q14.1		3q23	16q24.2		16p13.13	
	**rs2450140**	**rs11237477**	**rs11705932**	**rs3859027**	**rs12597021**	**rs2018199**	**rs2075158**
GAB2	0.909	0.7395					
USP35	0.1896	0.04572					
TFDP2			0.3403				
ATP1B3			0.7273				
ZFPM1				0.2993	0.1304		
BCAR4						0.2793	0.1407
GSPT1						0.00059	0.00051
TNFRSF17						0.5626	0.4104
RSL1D1						0.05329	0.0431

Supplementary table 2 - TGCT predisposition loci identified to date, presented in chronological order with functional grouping based on previous review paper[3]. For loci with multiple reported SNPs the marker listed is taken from first study in the reference column.

**SNP**	**Locus**	**Candidate Gene(s)**	**Functional Grouping**	**Reference(s)**
rs995030	12q21	*KITLG*	1) *KIT/KITLG* signalling	4,5
rs210138	6p21	*BAK1*	1) *KIT/KITLG* signalling<br>2) Apoptosis	4,5
rs4624820	5q31	*SPRY4*	1) *KIT/KITLG* signalling	4
rs4635969	5p15	*TERT*<br>*CLPTM1L*	3) Telomerase function<br>2) Apoptosis	6
rs755383	9p24	*DMRT1*	7) Sex determination	6
rs2900333	12p13	*ATF7IP*	3) Telomerase function	6,7
rs8046148	16q12.1	*HEATR3*	unknown/other	8
rs2839243	21q22.3	*non-coding*	unknown/other	8
rs3805663	5q31.1	*CATSPER3*<br>*PITX1*	unknown/other<br>3) Telomerase function	8
rs10510452	3p24.3	*DAZL*	4) Male germ cell development	8
rs2720460	4q24	*CENPE*	5) Centrosome cycle	8

rs7010162	8q13.3	*PRDM14*	4) Male germ cell development	8
rs9905704	17q22	*RAD51C*	6) DNA Repair	9
		PPM1E	unknown/other	
		TEX14	5) Centrosome cycle	
rs3790672	1q24.1	non-coding	unknown/other	8,10
rs2072499	1q22	*PMF1*	5) Centrosome cycle	8,9
rs4888262[c]	16q22.3	*RFWD3*	6) DNA Repair	9
rs12699477	7p22.3	*MAD1L1*	5) Centrosome cycle	9
rs17021463	4q22.2	*HPGDS*	4) Male germ cell development	9
rs1510272	3q25	*SSR3*	unknown/other	11
		TIPARP	unknown/other	
rs7501939	17q12	*HNF1B*	unknown/other	12
rs2195987	19p12	non-coding	unknown/other	12
rs11705932	3q24	*TFDP2*	unknown/other	new
		ATP1B3	unknown/other	
rs7107174	11q14	*GAB2*	1) *KIT/KITLG* signalling	new
		USP35	unknown/other	
rs4561483	16p13	*BCAR4*	unknown/other	new
		RSL1D1	unknown/other	
		GSPT1	2) Apoptosis	
		TNFRSF17	unknown/other	
rs55637647	16q24	*ZFPM1*	7) Sex determination	new

Supplementary table 3 – Pathway associations with FDR < 0.1

**Pathway/Gene set name**	***P*-value**	**False Discovery Rate**
CELLULAR COMPONENT DISASSEMBLY	< 0.001	0.0210
Sex determination gene set (Custom Set)	< 0.001	0.0252
CENTROSOME CYCLE	< 0.001	0.0375
INDUCTION OF APOPTOSIS BY INTRACELLULAR SIGNALS	< 0.001	0.0706
KIT/KITLG Signalling (Custom Set)	< 0.001	0.0729
PROTEIN COMPLEX DISASSEMBLY	< 0.001	0.0738
MACROMOLECULAR COMPLEX DISASSEMBLY	< 0.001	0.0738
CONDENSED CHROMOSOME	0.001	0.0784
SINGLE STRANDED RNA BINDING	< 0.001	0.0796
TRANSCRIPTION FROM RNA POLYMERASE III PROMOTER	0.001	

		0.0798
CHROMOSOME	0.003	0.0806
TRANSITION METAL ION BINDING	0.004	0.0806
INTRA GOLGI VESICLE MEDIATED TRANSPORT	0.001	0.0806
ZINC ION BINDING	0.001	0.0808
CELLULAR PROTEIN COMPLEX DISASSEMBLY	< 0.001	0.0812
NKCELLSPATHWAY	< 0.001	0.0823
DNA DAMAGE RESPONSE SIGNAL TRANSDUCTION BY P53 CLASS MEDIATOR	< 0.001	0.0826
HSA04120 UBIQUITIN MEDIATED PROTEOLYSIS	< 0.001	0.0826
IL17PATHWAY	0.001	0.0837
RESPONSE TO ABIOTIC STIMULUS	0.001	0.0840
RESPONSE TO IONIZING RADIATION	0.001	0.0855
SMALL PROTEIN CONJUGATING ENZYME ACTIVITY	< 0.001	0.0871
DNA DAMAGE RESPONSE SIGNAL TRANSDUCTION RESULTING IN INDUCTION OF APOPTOSIS	< 0.001	0.0873
HYPERTROPHY MODEL	0.001	0.0878
MANNOSYLTRANSFERASE ACTIVITY	< 0.001	0.0891
CELLULAR PROTEIN METABOLIC PROCESS	< 0.001	0.0901
CELLULAR MACROMOLECULE METABOLIC PROCESS	< 0.001	0.0921
G2PATHWAY	0.002	0.0928
DNA DAMAGE RESPONSE SIGNAL TRANSDUCTION	0.002	0.0935
IGF1PATHWAY	0.001	0.0991
RESPONSE TO RADIATION	< 0.001	0.0997

Supplementary Note

Supplementary note 1: The UK Testicular Cancer Collaboration (UKTCC)

Principal Investigator	Study Centre	Study centre address
Rustin, Prof Gordon	Mount Vernon Hospital	Mount Vernon Cancer Centre, Rickmansworth Road, Northwood, Middlesex, HA6 2RN
Srihari, Dr	Royal Shrewsbury Hospital	Trials Unit, Oncology Department, Mytton Oak Road, Shrewsbury, SY3 8XB
Cole, Dr David	Great Western Hospital	3rd Floor, Osprey Unit, Swindon, Wilts, SN3 6BB
Askill, Dr Colin & Bertelli, Dr Gianfilippo	Singleton Hospital and Morriston Hospital	SWW Cancer Institute, Sketty, Swansea, SA2 8QA
Barber, Dr James	Velindre Hospital	Clinical Trials Unit, Velindre Cancer Centre, Velindre Road, Whitchurch, Cardiff CF14 2TL
Gilby, Dr Ed	Royal United Hospital	Dept of Oncology and Haematology, Combe Park, Bath, BA1 3NG
Huddart, Dr Robert	Royal Marsden Hospital Sutton	Downs Rd, Sutton, SM2 5PT
White, Dr Jeff	Beatson Oncology Centre	Beatson West of Scotland Cancer Centre, 1053 Great Western Road, Glasgow, G11 0YN
Braybrooke, Dr Jeremy	Bristol Haematology & Oncology Centre	United Bristol Healthcare NHS trust, Horfield Rd, Bristol, BS2 8ED
Leahy, Dr M and Welch, Dr R	Christie Hospital	Wilmslow Road, Withington, Manchester, M20 4BX
Chakraborti, Dr P	Derbyshire Royal Infirmary	Derby Hospitals NHS Trust, London Road, Derby, DE1 2QY
Joffe, Dr J	St James Hospital Leeds	Dept of Medical Oncology, Leeds, LS9 7TF

Brown, Dr Richard	Wexham Park Hospital	Cancer Clinical Trials, John Ulster Post Grad Centre, Slough, Berks, SL2 4HL
Faust, Dr Guy	Leicester Royal Infirmary	LNR Cancer Reseach Network, Knighton St, Leicester LE1 5WW
Simmonds, Dr Peter	Southampton General Hospital	Cancer Care Directorate, Medical Oncology, Mailpoint 306, Southampton General Hospital, Tremona Rd, SO16 6YD
Mazhar, Dr danish	Addenbrookes Hospital	Addenbrookes Hospital, Cambridge Clinical Trials Centre, Oncology Clinical Trials, (S4) Box 279, Hills Rd, CB2 0QQ
Stockdale, Dr A & Hrouda, Dr D & Humber, Dr C.	University Hospital Walsgrave	Arden Cancer Centre, West Wing, UHCW NHS trust, Clifford Bridge Rd, Coventry, CV2 2DX
Appel, Dr Wiebke	Royal Preston Hospital	Dept of Oncology, Royal Preston Hospital, Sharoe Green Lane North, Fulwood Preston, PR2 9HT
Hong, Dr Anne	Royal Devon & Exeter	Exeter Oncology Centre, Royal Devon and Exeter Hospital, Barrack Rd, Exeter EX2 5DW
Dr Howard	Western General Hospital	Scottish Cancer Research Network, Oncology Admin Corridor, Edinburgh Cancer Centre, Western General Hospital, Crewe Rd South, Edinburgh, EH4 2XU
Dr Fiona Douglas	Freeman Hospital	Clinical Trials Unit, Newcastle General Hospital, Westgate Rd, Newcastle-upon Tyne, NE4 6BE
Bllomfield, Dr David	Royal Sussex County Hospital	Brighton and Sussex University Hospitals, The Sussex Cancer Centre, The Royal Sussex County Hospital, Eastern Road, Brighton, BN2 5BE
Dr Mohammad Butt	Castle Hill Hospital	Castle Hill Hospital, Castle Road, Cottingham HU16 5JQ
Dr Kay Kelly	Raigmore Hospital	Raigmore Hospital, Old Perth Road, Inverness, IV2 3UJ

Dr R Mehra	New Cross Hospital	Greater Midlands Cancer Research Network, The Chestnuts, The Royal Wolverhampton Hospitals, New Cross Hospital NHS Trust, Wednesfield Road, Wolverhampton, WV10 0QP
Dr Richard Brown/Dr Paul Rogers	Royal Berkshire Hospital	Royal Berkshire Hospital, Berkshire Cancer Centre, London Road, Reading, Berkshire, RG1 5AN
Chakraborti, Dr P	Queen's Hospital Burton	Queens Hospital, Burton upon Trent, Belvedere Road, Burton, DE13 0RB
Dr Matthew Hatton	Weston Park Hospital	Consultant Clinical Radiologist. Sheffield Teaching Hospitals NHS Foundation Trust, 8 Beech Hill Road, Sheffield S10 2SB
Hennig, Dr Ivo	Nottingham City Hospital	Nottingham University Hospitals NHS Trust, City Hospital campus, Hucknall Road, Nottingham, NG5 1PB
Dr J McAteer	Belfast City Hospital	Northern Ireland Cancer Centre, Belfast City Hospital, Lisburn Rd, Belfast, BT9 7AB
Dr Savage/Dr Seckl	Charing Cross Hospital	Dept of Medical Oncology,Charing Cross, Fulham, Palace Rd, London W6 8RF
Dr Joanna Gale	Portsmouth Haematology & Oncology Centre	Level B Queen Alexandra Hospital, Cosham, Portsmouth, PO6 3LY
Rustin, Prof Gordon	Hillingdon Hospital	R&D Office - Education Centre, Hillingdon Hospital, Pield Heath Road, Hillingdon, UB8 3NN
Prof Peter Clark	Royal Liverpool & Broadgreen Hospitals	Prescot Street Liverpool, L78XP
Dr Steve Woby	Royal Oldham Hospital/Pennine Acute Hospital	Roachdale Road Oldham OL1 2JH
Dr Adrian Rathmell	James Cook Hospital	Middlesbrough TS4 3BW

Dr Alan Lamont	Colchester/Essex County Hospital	Essex County
Dr Guy Faust	Northampton General	Cliftonville, Northampton NN1 5BD
Dr Naveed Sarwar	Basildon Hospital	Nethermayne Basildon Essex SS16 5NL
Prof Nick Stuart	Glan Clwyd Hospital and Ysbyty Gwynedd	NW Cancer Treatment Centre, Glan Clwyd Hospital, LL18 5UJ
Dr Simon Chowdhury	Guys & St Thomas's	St Thomas Street, London SE1 9RT
Dr Sharon Beesley	Maidstone and Tunbridge NHS Trust	Maidstone Hospital, Hemitage Lane, Barming, Maidstone, Kent ME16 9QQ
Dr Winkler	West Middlesex University Hospital	West Middlesex University Hospital NHS Trust, R&D Department, 4th Floor, East Wing,Twickenham Road, Isleworth Middlesex TW7 6AF
Dr Abdel Hamid	Broomfield Hospital	Broomfield Hospital, West Wing 2, Court Road, Broomfield, Chelmsford, Essex CM1 7ET
Dr Sanjeev Pathak	Doncaster Royal Infirmary	Joint Research Office of Doncaster and Bassetlaw Hospitals NHS Foundation Trust, First Floor 'C' Block, Doncaster Royal Infirmary, Armthorpe Road, Doncaster DN2 5LT
Dr Krishnaswamy Madhavan	Southend University Hospital NHS Foundation Trust	Pittlewell Chase, Westcliff-On-Sea, Essex SSO 0RH
Dr Martin Highley	Derriford Hospital (Plymouth)	Plymouth Hospitals NHS Trust, Derriford Hospital, Plymouth, PL6 8DH
Dr Julian Money-Kyrle	Royal Surrey County Hospital	Royal Surrey County Hospital, St Lukes Cancer Centre, Egerton Road, Guildford, Surrey GU2 7XX
Dr Cathryn Brock	Chelsea & Westminster Hospital NHS Foundation Trust	Chelsea & Westminster Hospital, Unit 101, 1st Floor, Harbour Yard, Chelsea Harbour, London SW10 0XD
Dr Thiagarajan Sreenivasan	United Linconshire Hospitals NHS Trust	Lincoln County Hospital, Greetwell Road, Lincoln, LN2 5QY

Dr Thiagarajan Sreenivasan	United Linconshire Hospitals<br><br>NHS Trust	Pilgrim Hospital, Boston, Lincolnshire PE21 9QS

Supplementary References

1. Koster, R. *et al.* Pathway-based analysis of GWAs data identifies association of sex determination genes with susceptibility to testicular germ cell tumors. *Hum Mol Genet* (2014).
2. Szklarczyk, D. *et al.* STRING v10: protein-protein interaction networks, integrated over the tree of life. *Nucleic Acids Res* **43**, D447-52 (2015).
3. Litchfield, K., Shipley, J. & Turnbull, C. Common variants identified in genome-wide association studies of testicular germ cell tumour: an update, biological insights and clinical application. *Andrology* **3**, 34-46 (2015).
4. Rapley, E.A. *et al.* A genome-wide association study of testicular germ cell tumor. *Nat Genet* **41**, 807-10 (2009).
5. Kanetsky, P.A. *et al.* Common variation in KITLG and at 5q31.3 predisposes to testicular germ cell cancer. *Nat Genet* **41**, 811-5 (2009).
6. Turnbull, C. *et al.* Variants near DMRT1, TERT and ATF7IP are associated with testicular germ cell cancer. *Nat Genet* **42**, 604-7 (2010).
7. Kanetsky, P.A. *et al.* A second independent locus within DMRT1 is associated with testicular germ cell tumor susceptibility. *Hum Mol Genet* **20**, 3109-17 (2011).
8. Ruark, E. *et al.* Identification of nine new susceptibility loci for testicular cancer, including variants near DAZL and PRDM14. *Nat Genet* **45**, 686-9 (2013).
9. Chung, C.C. *et al.* Meta-analysis identifies four new loci associated with testicular germ cell tumor. *Nat Genet* **45**, 680-5 (2013).
10. Schumacher, F.R. *et al.* Testicular germ cell tumor susceptibility associated with the UCK2 locus on chromosome 1q23. *Hum Mol Genet* **22**, 2748-53 (2013).
11. Litchfield, K. *et al.* Multi-stage genome wide association study identifies new susceptibility locus for testicular germ cell tumour on chromosome 3q25. *Hum Mol Genet* (2014).
12. Kristiansen, W. Two new loci and gene sets related to sex determination and cancer progression are associated with susceptibility to testicular germ cell tumour. *Human Molecular Genetics (In press)* (2015).
